# Does the Saudi Population Have Sufficient Awareness of Vitiligo in Southwest Saudi Arabia? A Cross-Sectional Survey, 2022

**DOI:** 10.3390/clinpract12060092

**Published:** 2022-11-04

**Authors:** Fatmah Ahmed Keraryi, Alhanouf Adel Hadi Hakami, Nouf Adel Hadi Hakami, Mohamed Salih Mahfouz, Hadi Adel Hadi Hakami

**Affiliations:** 1 Department of Dermatology, Sabya General Hospital, Ministry of Health, Jazan 45142, Saudi Arabia; 2 Jazan General Hospital, Ministry of Health, Jazan 45142, Saudi Arabia; 3 King Fahd Central Hospital, Ministry of Health, Jazan 45142, Saudi Arabia; 4 Department of Family and Community Medicine, Faculty of Medicine, Jazan University, Jazan 45142, Saudi Arabia; 5 Faculty of Medicine, Jazan University, Jazan 45142, Saudi Arabia

**Keywords:** vitiligo, skin condition, knowledge, attitudes, Jazan

## Abstract

Background: Vitiligo is a common acquired hypopigmentation skin condition defined by an idiopathic, gradual, and restricted loss of melanin pigment from otherwise healthy-looking skin. The present study aims to evaluate the knowledge and perceptions of and attitudes toward vitiligo among the general population of Jazan Province, Saudi Arabia. Methods: An observational cross-sectional survey targeting adults of the Jazan region was conducted in 2022. The data were collected using an electronic questionnaire distributed through social media in the region. Statistical analysis was performed using the SPSS computer program. Knowledge and attitude scores were computed. Results: Most of the respondents (83.7%) had an adequate knowledge level of vitiligo, and almost half of the respondents (46.8%) had a positive attitude toward the disease. The majority of the respondents (84.5%) knew that vitiligo is not contagious. However, the majority of the participants (78.6%) did not know that vitiligo is an autoimmune disease. Most of the respondents (93.1%) do not avoid shaking hands with a vitiligo patient to prevent infection, accept food prepared by a vitiligo patient (69.4%), accept sharing a plate with a vitiligo patient (73.6%), and accept to be helped by a vitiligo patient (86.7%). However, about half of the participants (50.4%) refused to marry a person diagnosed with vitiligo. The knowledge of vitiligo in the study population was not correlated with any of the demographic characters, such as age, gender, education, or occupation (*p* > 0.05). The most important factors associated with good attitudes were male gender (COR = 1.92; 95% CI: 1.92–2.84; *p* = 0.001) and being aged over 50 years (COR = 3.06; 95% CI: 1.53–6.13; *p* = 0.002). Conclusions: The population in Jazan had a good level of knowledge of vitiligo and a positive attitude toward patients with vitiligo. Social media is the source of information for vitiligo for the majority of the study population. The good attitude toward patients with vitiligo was more prevalent in males and older people. More efforts are needed to improve the knowledge of and attitudes toward vitiligo.

## 1. Introduction

Vitiligo is a common acquired hypopigmentation skin disorder characterized by an idiopathic, progressive, and circumscribed loss of melanin pigment from otherwise healthy-looking skin, with a complete absence of functioning melanocytes in microscopic examination [1,2,3,4]. The distribution of macules may be localized or generalized and may coalesce into wide, depigmented areas. These macules are usually cosmetically disfiguring, especially in individuals with dark skin. Skin lesions are often visible to others, so vitiligo is commonly associated with emotional distress, social stigma, and poor quality of life [5,6,7].

The literature suggests that vitiligo is the most common cause of skin depigmentation, with a worldwide incidence rate ranging from 0.1% to 2% [1,8,9]. The prevalence of vitiligo is equally distributed in males and females and individuals from different racial, ethnic, or socioeconomic backgrounds [4]. Vitiligo may occur at any age, but its incidence peaks in the second and third decades. Among patients with vitiligo, approximately 33% are children, and 70% to 80% are adults before the age of 30 years [3]. The etiology of vitiligo is not fully understood, but seems to include an interaction of genetic, neurological, and immunological factors [1,10]. Patients commonly attribute the onset of their disease to specific events, such as physical injury, pregnancy, sunburn, or emotional stress. However, at present, there is no evidence supporting a causative relationship of these factors with vitiligo. The high frequency of comorbid autoimmune diseases, such as halo nevus and malignant melanoma, in patients with vitiligo suggests an autoimmune etiology of the disease [4].

Several observational studies have examined the knowledge of and attitudes toward vitiligo and showed that misconceptions and poor awareness of vitiligo are prevalent among the public and even patients themselves [9,10,11,12,13,14]. Although a few studies [13,14,15] have been conducted assessing the knowledge of and attitude toward vitiligo in the Kingdom of Saudi Arabia (KSA), no study has been conducted in Jazan, southwest Saudi Arabia. The present study aims to evaluate the knowledge and perceptions of and attitudes toward vitiligo among the general population of Jazan Province, Saudi Arabia, and to explore the local myths.

## 2. Materials and Methods

### 2.1. Ethical Considerations

This study was conducted in accordance with the ethical standards within the political borders of the KSA and Saudi bioethics and the Declaration of Helsinki. All of the participants read, understood, and signed a written consent form. The study was approved by Jazan Health Ethics Committee, Approval No. 2284. The participants were told that they have the freedom to participate or to withdraw from the study at any time. 

### 2.2. Study Design, Setting, and Population

To examine the knowledge of and attitude toward vitiligo among the general population, a descriptive cross-sectional study was used. This study was conducted in Jazan, southwest Saudi Arabia. The region is the second smallest region in the country and covers an area of 11,671 km^2^ with a population of 1,567,547. The study included Saudi adults aged 18 years or older who were willing to take part in the study. Non-Saudi and younger adults were excluded.

### 2.3. Sampling Procedure

The sample size was calculated using the Raosoft website. A sample of 480 participants was estimated for this study; the sample size was calculated using the sample size formula n = Z21-α P (1 − P)/d2. The following parameters were utilized to calculate the sample size: prevalence of knowledge P = 50%, confidence interval Z = 95%, error d = not more than 5%, and a 20% non-response rate (online surveys require a large sample size as the non-response rate is always high). The sampling plan utilized the administrative distribution of the Jazan region, with the survey link distributed in each sub-administration unit.

### 2.4. Data Collection and Study Tool

Questionnaires were designed and sent to experts for content validity before being administered to the participants. The questionnaires used were standardized for consistency and for reliability and validity purposes in the quantitative analysis. The data were collected via an online self-administered questionnaire that was sent to the sample of participants selected through social media platforms. The questionnaire items were adopted from a previously prepared self-structured validated questionnaire [14]. The questionnaire included questions related to the sociodemographic characteristics of the participants. Knowledge and perceptions of and attitudes toward vitiligo were assessed using 17 items in three main categories. Regarding the knowledge items, a score of 10 was given to each correct response, with incorrect responses being scored 0. A total knowledge score was computed by summing the individual responses for each participant. Adequate knowledge was defined as a total score above 50% of the correct responses. Lower scores indicated inadequate knowledge. Regarding the attitude items, a score of 10 was given for each positive attitude, and 0 for a negative attitude. A total attitude score was computed by summing the individual responses for each participant. Participants with scores over 50% were classified as having a good attitude. The tool’s reliability was assessed using Cronbach’s alpha and revealed a value of 0.60.

### 2.5. Data Analysis 

The computer program IBM SPSS Statistics for Windows, version 24.0 (IBM Corp., Armonk, NY, USA), was used to enter, organize, tabulate, and analyze the data. The participants’ socioeconomic status and demographic data were tabulated, and the percentage and frequency were calculated. The chi-square test was used to test the significance of the association of the stated variables. Multivariate logistic regression analysis was used to assess the independent predictors among the participants. A *p*-value of less than 0.05 was considered statistically significant.

## 3. Results

A total of 496 participants were approved to fill in a self-administered online questionnaire assessing the knowledge of and attitude toward vitiligo in the Jazan district in the KSA. Table 1 shows that the study included participants of different ages; about half (49.4%) were between 31 and 50 years old. Participants with a bachelor’s degree and secondary education reported higher knowledge scores (M = 71.89, SD = 16.7; M = 71.26, SD = 14.2) than the other participants, but without statistical significance (*p* > 0.05). Young participants (18–30 years) showed higher positive attitude scores (M = 56.13, SD = 20.3) than the other age groups, but also without statistical significance (*p* > 0.05).

Table 2 shows that most respondents (98.2%) had heard about vitiligo before. The majority of the respondents (84.5%) knew that vitiligo is not contagious. Additionally, 88.1% of the respondents answered “No” when asked whether vitiligo is related to the ingestion of fish or eggs. However, the majority of the participants (78.6%) did not know that vitiligo is an autoimmune disease, and 64.7% of the respondents reported that vitiligo is not a hereditary disease. In addition, 69.8% of them did not know that vitiligo worsens with psychological stress, and 69% of the study population reported that vitiligo affects the social life of patients. Most participants (95.4%) reported that vitiligo is a dangerous disease. About half of the respondents reported that there is no treatment for vitiligo.

**Figure 1:**
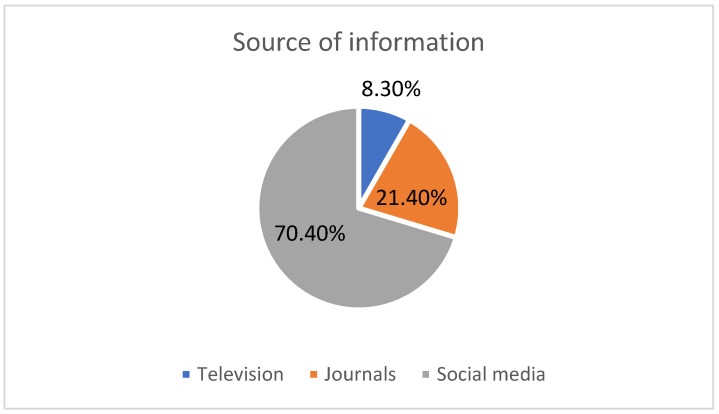
Source of information on vitiligo.

In Figure 1, the graph shows that social media is the main source of information about vitiligo for the majority of the study population (70.4%), followed by journals (21.4%) and TV (8.3%).

Table 3 shows that about half of the respondents (46%) reported that vitiligo is a common disease in the KSA. Most of the respondents (93.1%) do not avoid shaking hands with vitiligo patients to prevent infection, accept food prepared by vitiligo patients (69.4%), accept sharing plates with vitiligo patients (73.6%), and accept to be helped by a vitiligo patient (86.7%). The majority of the participants (76.6%) thought that there was a lack of awareness regarding vitiligo.

Table 4 shows the association between demographic factors and knowledge status. More males than females had a high level of knowledge (83.7% compared with 80.8%), but without statistical significance (*p* > 0.05). The table further indicates that the knowledge of vitiligo in the study population was not correlated with any of the demographic characteristics, such as age, education, or occupation (*p* > 0.05 for all). 

Table 5 shows that the participants’ attitude toward vitiligo is significantly associated with age (*p* = 0.004) and gender (*p* = 0.001). However, the participants’ education level and occupation have no significant association (*p* > 0.05) with the attitude toward vitiligo.

The logistic regression analyses for the factors associated with positive attitudes toward vitiligo are presented in Table 6. The table revealed that the most important factors associated with good attitudes were male gender (COR = 1.92; 95% CI: 1.92–2.84; *p* = 0.001) and an age of more than 50 years (COR = 3.06; 95% CI: 1.53–6.13; *p* = 0.002).

## 4. Discussion

Vitiligo, a widespread depigmenting skin condition, is estimated to affect between 0.1% and 2% of the global population [14]. The condition is distinguished by a selective loss of melanocytes, resulting in non-scaly, chalky-white macules [15]. Significant progress has been made in the knowledge of vitiligo etiology, which is now defined as an autoimmune disease. Vitiligo is sometimes ignored as a cosmetic issue, although its symptoms can be psychologically distressing and have a significant impact on everyday life [16].

The emotional impact of vitiligo is significant and well documented [17,18]. The skin is a crucial part of our connection with the environment, and noticeable skin problems can restrict healthy psychosocial development due to the stigma associated with these conditions. Historically, skin illnesses and the people who suffer from them have been stigmatized [19]. Therefore, the current study aims to evaluate the knowledge and perceptions of and attitudes toward vitiligo among the general population of Jazan Province, Saudi Arabia.

Our results showed that about half of the participants (49.4%) were aged between 31 and 50 years old. The majority of the respondents were males (69.6%) and had a bachelor’s degree. More than half (57.5%) of the participants were employed. A study from northern India by Mahajan et al. (2019) found that the onset age of vitiligo ranged from 6 months to 82 years, with the majority of patients (71.3%) experiencing the condition before the age of 25 [20]. Another study by El-Husseiny et al. (2021) also documented that the average onset age of vitiligo in children was 6.18 years, with a mean condition duration of 2.12 years [21]. A meta-analysis assessing the prevalence of vitiligo by Zhang et al. (2016) found that vitiligo was more common among females than among males [22].

In addition, our results showed that most of the respondents (98.2%) had heard about vitiligo before. The majority of the respondents (84.5%) knew that vitiligo is not contagious. Additionally, 88.1% of the respondents answered “No” when asked whether vitiligo is related to the ingestion of fish or eggs. In contrast, a study by Fatani et al. (2014) showed that only 6.9% of the respondents had heard of vitiligo [14]. A cross-sectional study by Juntongjin et al. (2018) agreed with our findings. The researchers stated that approximately two-thirds of the participants in the survey were aware that vitiligo does not transfer by direct touch [23]. Another Turkish study by Topal et al. (2016) also noted that 90% of the respondents believed that vitiligo was not contagious [24].

The present study also illustrated that the majority of the participants (78.6%) did not know that vitiligo is an autoimmune disease, and 64.7% of the respondents reported that vitiligo is not a hereditary disease. In addition, 69.8% of them did not know that vitiligo worsens with psychological stress, and 69% of the study population reported that vitiligo affects the social life of patients. Most of the participants (95.4%) reported that vitiligo is a dangerous disease. About half of the respondents reported that there is no treatment for vitiligo. A study by Topal et al. (2016) showed that according to 84%, 37%, and 22% of the participants, respectively, stress, excessive sun exposure, and inheritance were thought to be causes of vitiligo, while 35% thought that vitiligo had no significant influence on the quality of life. Thirty-six percent thought that the condition was serious [24].

Moreover, our results indicated that social media is the source of information about vitiligo in the majority of the study population (70.4%), followed by journals (21.4%) and TV (8.3%). Algarni et al. (2021) also supported our results. They found that the most often-mentioned source of information (34.7%) was social media/Internet, followed by family/friends/acquaintances (30%) [25]. An Ethiopian study by Tsadik et al. (2020) also mentioned that friends and family were regarded as the most prevalent source of knowledge regarding vitiligo (70%) [26]. Our current results also showed that about half of the respondents (46%) reported that vitiligo is a common disease in the KSA. In contrast, research by Al Shammrie et al. (2017), who studied the pattern of skin diseases in Saudi Arabia, showed that vitiligo comprised only 7% of skin diseases [27].

In this study, the majority of the respondents (93.1%) do not avoid shaking hands with vitiligo patients to prevent infection, accept food prepared by vitiligo patients (69.4%), accept sharing a plate with vitiligo patients (73.6%), and accept to be helped by a vitiligo patient (86.7%). Most of the participants (94.9%) had no partners diagnosed with vitiligo. About half of the participants (50.4%) refused to marry a person diagnosed with vitiligo. However, 89.1% of the study population thought that vitiligo does not affect marital life. The majority of the participants (76.6%) thought that there was a lack of awareness regarding vitiligo. A study by Alshammrie et al. (2019) also supported these findings, showing that the majority of the study participants (73.8%) would refuse to marry a vitiligo patient [28]. Tsadik et al. (2020) also found that 43.7% do not mind shaking hands with vitiligo patients, 39.3% would exchange meals with vitiligo patients, and 38.7% would receive food made by a vitiligo patient [26].

Our results indicated that the knowledge of vitiligo in the study population was not correlated with any of the demographic characteristics, such as age, gender, education, or occupation. Juntongjin et al. (2018) also showed that there were no statistically significant associations between the knowledge score and gender, age, marital status, occupation, education, family members working in the healthcare industry, and monthly income [23].

Furthermore, our results indicated that the attitude of the participants toward vitiligo was significantly associated with age and gender. However, the education level or occupation of the participants has no significant association with the attitude toward vitiligo. The attitude of the participants toward vitiligo is significantly associated with age. It was also found that a good attitude toward vitiligo is more often found in older participants than in younger ones. The attitude of the participants toward vitiligo was significantly associated with gender. A hospital-based study by Asati et al. (2016) supports these findings. The researchers found that males, younger age groups, married people, and those who work in healthcare or are jobless had considerably higher attitude scores. Female gender was the only other significant indicator with a low attitude score [11].

Our research has some limitations that should be reported. First, the study is an observational cross-sectional survey, so the associations revealed by this study should be interpreted with caution. Second, the study was conducted using a web-based survey, which decreases the generalization of the study outcomes.

## 5. Conclusions

In conclusion, our study found that the people of Jazan had a high level of knowledge of vitiligo and good attitudes toward vitiligo patients. The majority of the study population regarded social media as the primary source of knowledge of vitiligo. The majority of those surveyed were aware that vitiligo is not contagious. The study population’s knowledge of vitiligo was not associated with any of the demographic parameters, such as age, gender, education, or occupation. The participants’ attitudes toward vitiligo were substantially related to their age and gender. Males and older adults had a more positive attitude toward vitiligo patients.

## Figures and Tables

**Table 1:** Study participants’ background characteristics and knowledge and attitude scores according to some selected characteristics (*n* = 496).

Variables		*n*	%	Knowledge Scores		Attitude Scores	
				Mean	SD	Mean	SD
Age group (years)	18–30 years	208	41.9	72.48	(16.3)	56.13	(20.3)
	31–50 years	245	49.4	70.36	(15.8)	52.19	(21.2)
	>50 years	43	8.7	70.35	(15.2)	46.51	(20.7)
Gender	Male	345	69.6	71.70	(16.0)	51.56	(21.2)
	Female	151	30.4	70.20	(15.9)	57.45	(19.6)
Education level	Primary	6	1.2	64.58	(14.6)	58.33	(25.8)
	Preparatory	22	4.4	64.20	(18.2)	56.82	(18.0)
	Secondary	167	33.7	71.26	(14.2)	50.67	(23.5)
	Bachelor	301	60.7	71.89	(16.7)	54.49	(19.4)
Occupation	Unemployed	211	42.5	71.92	(16.3)	53.97	(20.7)
	Employed	285	57.5	70.75	(15.7)	52.89	(21.1)

No statistical differences were reported (*p* > 0.05 for all variables).

**Table 2:** Participants’ responses to questions regarding the knowledge of vitiligo.

Questions		Count	Percentage
Have you heard about vitiligo before?	No	9	1.8%
	Yes	487	98.2%
Is vitiligo contagious?	No	419	84.5%
	Yes	77	15.5%
Is vitiligo related to the ingestion of fish or eggs?	No	437	88.1%
	Yes	59	11.9%
Is vitiligo an autoimmune disease?	No	390	78.6%
	Yes	106	21.4%
Is vitiligo a hereditary disease?	No	321	64.7%
	Yes	175	35.3%
Does vitiligo worsen with psychological stress?	No	346	69.8%
	Yes	150	30.2%
Does vitiligo affect the social life of patients?	No	154	31.0%
	Yes	342	69.0%
Is vitiligo a dangerous disease?	No	473	95.4%
	Yes	23	4.6%
Is there a treatment for vitiligo?	No	258	52.0%
	Yes	238	48.0%

**Table 3:** Participants’ responses to questions regarding the attitudes toward vitiligo.

Questions		Count	Percentage
Is vitiligo a common disease in the KSA?	No	268	54.0%
	Yes	228	46.0%
Would you avoid shaking hands with a vitiligo patient to prevent infection?	No	462	93.1%
	Yes	34	6.9%
Would you eat food prepared by a vitiligo patient?	No	152	30.6%
	Yes	344	69.4%
Would you share your plate with a vitiligo patient?	No	131	26.4%
	Yes	365	73.6%
Would you accept to be helped by a vitiligo patient?	No	66	13.3%
	Yes	430	86.7%
Would you accept a vitiligo patient to be an heir?	No	84	16.9%
	Yes	412	83.1%
Would you marry a person diagnosed with vitiligo?	No	250	50.4%
	Yes	246	49.6%
Is your spouse diagnosed with vitiligo?	No	392	94.9%
	Yes	21	5.1%
If you are married, does vitiligo affect your marital life?	No	303	89.1%
	Yes	37	10.9%
Do you think that there is a lack of awareness regarding vitiligo?	No	116	23.4%
	Yes	380	76.6%

**Table 4:** Association between demographics and knowledge status.

Variables		Knowledge Level				*p*-Value
		Low		High		
		*n*	%	*n*	%	
Age group	18–30 years	34	(16.3)	174	(83.7)	0.099
	31–50 years	40	(16.3)	205	(83.7)	
	>50 years	7	(16.3)	36	(83.7)	
Gender	Male	52	(15.1)	293	(84.9)	0.252
	Female	29	(19.2)	122	(80.8)	
Education level	Primary	2	(33.3)	4	(66.7)	0.095
	Preparatory	7	(31.8)	15	(68.2)	
	Secondary	22	(13.2)	145	(86.8)	
	Bachelor	50	(16.6)	251	(83.4)	
Occupation	Unemployed	32	(15.2)	179	(84.8)	0.546
	Employed	49	(17.2)	236	(82.8)	
All participants		81	(16.3)	415	(83.7)	

**Table 5:** Association between demographics and participants’ attitude toward vitiligo.

Variables		Attitude Score				*p*-Value
		Poor Attitude		Good Attitude		
		*n*	%	*n*	%	
Age group	18–30 years	124	(59.6)	84	(40.4)	0.004
	31–50 years	126	(51.4)	119	(48.6)	
	>50 years	14	(32.6)	29	(67.4)	
Gender	Male	167	(48.4)	178	(51.6)	0.001
	Female	97	(64.2)	54	(35.8)	
Education level	Primary	4	(66.7)	2	(33.3)	0.507
	Preparatory	14	(63.6)	8	(36.4)	
	Secondary	83	(49.7)	84	(50.3)	
	Bachelor	163	(54.2)	138	(45.8)	
Occupation	Unemployed	120	(56.9)	91	(43.1)	0.161
	Employed	144	(50.5)	141	(49.5)	
All participants		246	(53.2)	232	(46.8)	

**Table 6:** Factors associated with positive attitudes toward vitiligo.

Variables		Predictors of Positive Attitudes			
		COR	95% CI		*p*-Value
			Lower	Upper	
Gender	Male (REF = Female)	1.92	1.29	2.84	0.001
Age group	18–30 years	REF			
	31–50 years	1.39	0.96	2.03	0.081
	>50 years	3.06	1.53	6.13	0.002
Educational level	Primary	REF			
	Preparatory	1.14	0.17	7.69	0.891
	Secondary	2.02	0.36	11.35	0.423
	Bachelor	1.69	0.31	9.38	0.547
Occupation	Employed (REF = Unemployed)	1.29	0.90	1.85	0.162

## Data Availability

Not applicable.
